# Drought drives selection for earlier flowering, while pollinators drive selection for larger flowers in annual *Brassica rapa*

**DOI:** 10.1093/aobpla/plae070

**Published:** 2025-01-08

**Authors:** Kaushalya Rathnayake, Amy L Parachnowitsch

**Affiliations:** Department of Biology, 10 Bailey Drive, University of New Brunswick, Fredericton, NB E3B 5A3, Canada; Department of Biology, 10 Bailey Drive, University of New Brunswick, Fredericton, NB E3B 5A3, Canada

**Keywords:** *Brassica rapa*, climate change, drought, flowering phenology, microevolution, nectar, plant–pollinator interactions, phenotypic plasticity, phenotypic selection

## Abstract

Drought-induced changes in floral traits can disrupt plant–pollinator interactions, influencing pollination and reproductive success. These phenotypic changes likely also affect natural selection on floral traits, yet phenotypic selection studies manipulating drought remain rare. We studied how drought impacts selection to understand the potential evolutionary consequences of drought on floral traits. We used a factorial experiment with potted plants to manipulate both water availability (well-watered and drought) and pollination (open and supplemented). We examined the treatment effects on traits of *Brassica rapa* and estimated phenotypic selection and whether it was pollinator-mediated in these two abiotic conditions. Drought affected plant phenotypes, leading to plants with fewer flowers and ultimately lower seed production. Flowering time did not show variation with watering, but we found the strongest effect of drought on selection was for flowering time. There was a selection for flowering faster in drought but not well-watered conditions. Pollinators instead were the agents responsible for selection on flower size, but we did not find strong evidence that drought effected pollinator-mediated selection. There was a stronger selection for larger flowers in drought compared to well-watered plants, and it could be attributed to pollinators however, there was no significant difference between watering treatments. Our results show the effects of drought are not limited to phenotypic responses and may alter evolution in plants by changing phenotypic selection on traits. The connection between phenotypic plasticity and selection may be important to understand as we found the most variable trait (display size) was not under selection while the trait with different selection in drought (flowering time) did not change in response to drought. Our study highlights the importance of manipulating potential agents of selection, especially to understand fully the potential impacts of components of climate change such as drought.

## Teaser

Using experimental populations of field mustard, we found that in drought there was natural selection for flowering early but pollinators selected for larger flowers regardless of watering conditions. Our results show that drought may not be limited to affecting a plant's condition but may also alter the evolution of floral traits. However, our experiment did not show strong evidence that drought altered how pollinator-mediated selection acted on floral traits.

## Introduction

Climate change has altered precipitation patterns across the globe, increasing the frequency and severity of summer droughts in many areas (Spinoni *et al.* 2014; Naumann *et al.* 2018). Drought can directly affect plant reproduction by lowering flower production (Burkle and Runyon, 2016; Descamps *et al.* 2020; Höfer *et al.* 2022) and increasing floral abortion rates (Fang *et al.* 2010; Descamps *et al.* 2018). Water stress can also impact plant fitness indirectly by influencing how pollinators interact with flowers, altering pollinator services and subsequent reproductive success (Descamps *et al.* 2018; Kuppler *et al.* 2021). Drought often leads to smaller plants, inflorescences, flowers, and reduced nectar rewards (Kuppler and Kotowska 2021), which could lead to disruptions in plant-pollinator interactions that can affect seed production, as is expected for global warming (Gérard *et al.* 2020). Therefore, studying the effect of water stress on floral traits and pollinator behaviour has been a major theme of ecological studies in recent years (Descamps *et al.* 2021; Kuppler and Kotowska 2021).

Changes to plant and floral phenotypes due to phenotypic plasticity with drought can have broader implications for evolution. First, changing trait distributions, such as smaller and/or short-lived flowers (Carroll *et al.* 2001; Burkle and Runyon 2016; Gallagher and Campbell 2017; Kuppler *et al.* 2021) can shrink the morphological trait variation in plant populations. Because natural selection acts on phenotypic variation, phenotypic plasticity, and genotype by environment interactions can influence the traits exposed to selection and evolution (Crispo 2008; Marais *et al.* 2013). Second, abiotic stresses such as drought could also affect the evolution of floral traits by lowering the population mean fitness, reducing the opportunity for selection (Rundle and Vamosi, 1996; Benkman 2013; Sletvold 2019). Thus to understand floral evolution in stressful environments, experiments that manipulate both abiotic factors and pollination are needed (Sletvold 2019). However, few studies have measured natural selection on floral traits in response to drought. Selection for earlier flowering time can differ with drought. For example, in *Arabidopsis thaliana*, selection for earlier flowering was stronger in drought (Brachi *et al.* 2012) while in *Avena barbata* it was in well-watered plants (Sherrard and Maherali 2006). There is also evidence that a naturally occurring drought has led to earlier flowering in *Brassica rapa* (Franks *et al.* 2007). While drought conditions are likely to change floral traits beyond flowering time, only two studies have measured the selection of other floral characters in drought conditions. García *et al* (2023) compared selection in potted plants that were assigned to water deficit (open pollination, drought), pollinator restriction (bagged flowers, well-watered), or control (open pollination, well-watered). They found selection for larger *Ipomea purpurea* flowers with less nectar in drought conditions but interestingly the selection could not be attributed to pollinators. In *Primula tibetica,* Wu *et al.* used natural variation in soil water content between two sites to compare pollinator-meditated selection and found some differences in selection for plant height, number of flowers, and corolla size with soil water (Wu *et al.* 2022, 2023). While these studies show that different outcomes are probable depending on species and context, it is important to manipulate conditions to understand the interactive effects of biotic and abiotic agents on the evolution of floral traits (Caruso *et al.* 2019; Sletvold 2019).

We investigated how water stress affects floral traits and female fitness in fast-cycling *Brassica rapa.* We then measured differences in phenotypic selection between well-watered and drought conditions and whether the selection was pollinator-mediated via female fitness. We manipulated water availability (well-watered and drought) and pollination method (open vs. supplemental hand pollination) using a common garden factorial experiment at a rooftop greenhouse where plants were moved outside on non-raining days for pollinator access. We assessed phenotypic traits in the four combinations of water availability and pollination method and asked the following questions: (i) How do traits of *Brassica rapa* change in response to reduced water availability? (ii) Does selection differ between well-watered and drought conditions? (iii) Can selection be attributed to pollinators? And (iv) Does pollinator-mediated selection differ between well-watered and drought conditions?

## Materials and methods

### Study species

We used ‘Standard’ rapid cycling *Brassica rapa* L (syn. *B. campestris*: Brassicaceae) from the Wisconsin Fast Plants^®^ Program (Carolina Biological Supply Company, Burlington, NC, USA). *Brassica rapa* is an annual plant with a short generation time of approximately 30–45 days from seed to developing fruits (Williams and Hill, 1986). These plants maintain sufficient genetic and phenotypic variability for microevolutionary studies (Ågren and Schemske 1992; Gervasi and Schiestl 2017; Zu *et al.* 2020) and despite their fast lifecycle, they can experience strong pollinator-mediated selection in experimental settings (Gervasi and Schiestl 2017; Zu *et al.* 2020; Dorey and Schiestl 2022, 2024). *Brassica rapa* has a gametophytic self-incompatibility system that limits inbreeding (Nasrallah and Nasrallah 1989; Waller *et al.* 2008) and a generalized pollination system (including bumblebees, honeybees, hoverflies, and butterflies) which makes them amenable for common garden experiments. There is also evidence that wild *B. rapa* populations evolve rapidly in response to drought (Franks *et al.* 2016; Johnson *et al.* 2022), suggesting that phenotypic selection would differ between well-watered and drought conditions.

### Study design

**Figure 1.**
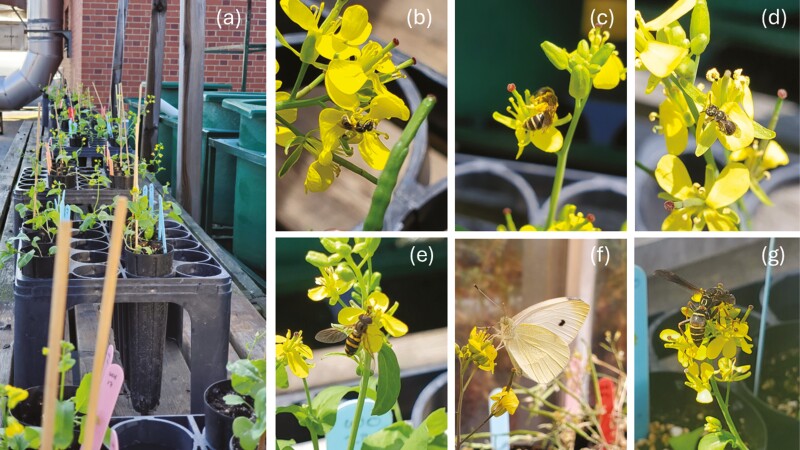
Outdoor exposure of experimental plants to visitors (A) and examples of visitors: sweat bees, *Lasioglossum sp.* (B)*, Halictus ligatus* (C), and *Lasioglossum sp.* (D), syrphid fly, *Eupeodes sp.* (E), cabbage butterfly, *Pieris rapae* (F), and paper wasp, *Polistes fuscatus* (G). Photographs by K. Rathnayake.

To help time the management of data collection and experimental watering and pollination conditions for 400 plants, we used a block design, planting over five consecutive weeks (15 June 2021 to 15 July 2021). Each week we sowed 2 *B. rapa* seeds in 84 Deepots^TM^ (635 ml, Stuewe & Sons, Inc.) at a rooftop greenhouse (Bailey Hall, University of New Brunswick) with supplemental light (6:00–20:00) and chillers to keep temperatures under ~25°C. Seeds were soaked for 12 hours prior to sowing, and we used VPW30 horticulture basic mix (ASB-Greenworld Inc.) with added Osmocote^®^ 14-14-14 controlled release fertilizer (2.1 kg m^-3^). With the first true leaves, we thinned to one plant per pot and arranged pots in two rows of five separated by an empty row in Deepot trays (Fig. 1). For each cohort, plants were watered every 1–2 days for the first 10 days, then the 80 plants were randomly assigned to either well-watered or drought (*N* = 40/treatment) and the remaining four plants were used only as pollen donors. Well-watered plants (W) and pollen donors received 100 ml water every 1–2 days. Watering of the drought plants (D) was based on the % soil moisture of haphazardly selected plants measured with an SM 150 soil moisture kit (Delta-Y devices, Cambridge, UK). When soil moisture reached roughly less than 50% of the well-watered pots (~3–6-day intervals), all drought plants were given 50 ml water. Soil moisture was measured for all plants at peak flowering and we confirmed the difference in percent soil moisture (mean ± SD) between treatments (well-watered: 34.21 ± 5.92, drought: 18.59 ± 7.78) with a linear model that included watering, pollination, their interaction, and cohort to account for differences among plantings. There was a significant effect of watering treatment (*F*_1_,_353_* *= 312.72, *P* < .00001) and cohort (*F*_4_,_353_* *= 27.26, *P* < .00001). We also confirmed that pollination treatments had similar soil moisture: no significant difference with pollination treatments (*F*_1_,_353_* *= 0.77, *P* = .39) or treatment interaction (*F*_1_,_353_* *= 0.76, *P* = .38).

To assess pollinator-mediated selection, when plants started flowering we randomly assigned 20 plants from each water treatment to either supplemental hand pollination (H) or open pollination only (O), giving four treatment groups: WO, WH, DO, and DH. To ensure there was sufficient pollen for all recipients, each day we randomly chose 10 plants to receive supplemental pollen from one of the four donor plants. Hand pollination was done by touching a pollination wand (Carolina Biological Supply Company) to fresh donor flowers and directly applying it to all open recipient flowers. To reduce the possibility of gametic incompatibilities, we then supplemented with additional pollen from the other donor plants and randomize the primary donor each day ensuring recipients had an equal chance to receive pollen from all four donor plants. Supplemental hand pollination was done prior to moving all trays with flowering plants to the rooftop just outside the greenhouse (Fig. 1) and flowers that were open for more than one day would receive multiple hand pollinations. Plants were outside each day without rain for a short period (from ~0700h to 1100h) to increase the likelihood of pollen limitation and detecting pollinator-mediated selection (Trunschke *et al.* 2017) and then returned to the greenhouse. Supplemental pollination and outdoor exposure occurred from the first flower until all plants in a cohort were done flowering. During the 2 months of the experiment, there were few days with morning rain that interfered with exposing plants to pollinators and all cohorts were outside for at least six days. Final watering occurred for the last cohort on 13 August 2021, after which all plants had senesced.

### Measurements

We recorded the date and number of first open flowers to assess phenology and calculated days to flower by subtracting the seeding date for each cohort. We counted the number of flowers (display size) at each plant’s peak flowering and measured petal length, flower diameter (flower size), the longest stamen, and pistil length to the nearest 0.01mm on three flowers per plant (when possible). Mean values for flower measures were used in analyses. We calculated herkogamy as pistil—stamen, which can affect pollen receipt in *B. rapa* (Syafaruddin *et al.* 2006). To measure nectar volume, we used 1µl micropipettes (Drummond Microcaps®, USA), pooled nectar from 2–3 flowers because nectar quantities are small for this species (Gervasi and Schiestl 2017; Dorey and Schiestl 2022) and divided by flower number for nectar volume/flower. We measured nectar concentration by diluting samples to provide sufficient fluid to use a hand-held digital refractometer (Palm Abbe^TM^, PA201, Misco, USA). However many were too diluted to get accurate readings, therefore we present only the nectar volume. All nectar measurements were taken prior to moving plants outside each day, although depending on flower opening these were likely a mix of new and pollinated flowers from the previous day. At the onset of fruit development, we measured plant height from the soil to the top of the plant using a ruler to the nearest 0.1 cm. To quantify female reproductive fitness, we counted fruits at maturation, which we harvested and dried to count total seeds.

### Data analysis

While all but two plants flowered in our experiment, we had uneven data collection across traits (see Results and online supplementary material). We tested the effects of our watering and pollination treatments and their interaction on traits (height, days to flower, display size, petal length, flower size, herkogamy, nectar volume) and female reproductive success (fruit number and total seeds) first with a multivariate analysis of variance (MANOVA). We included cohort as a blocking factor to control for differences in planting time (e.g. moisture, see above) here and in all subsequent analyses. We then conducted generalized linear models for individual traits with cohort as blocking factor. Mixed models found similar patterns, but we had reasonably few cohorts (5) for a random effect therefore we present the non-mixed model approach. We used a quasi-Poisson distribution for display size, nectar volume, fruits, and seeds to meet assumptions and otherwise used a normal distribution with the identity-link function.

We planned to calculate pollen limitation by comparing seed sets between open and supplemental pollinated treatments (Knight *et al.* 2005; Bennett *et al.* 2018). However, because supplemental pollination did not increase the seed set, we had little evidence of pollen limitation (see Results), making these estimates uninformative. We compared the opportunity for selection across watering and pollination treatments by estimating the variance of relative female fitness, as in other phenotypic selection studies (Sletvold *et al.* 2017; García *et al.* 2023; Wu *et al.* 2023).

To estimate selection, we included only plants with values for all included traits and fitness, which reduced the available plants in our treatments due to missing values (*N *= 89 well-watered/open-pollinated, 92 well-watered/hand-pollinated, 75 drought/open-pollinated, and 82 drought/hand-pollinated). We estimated directional selection (selection gradients, β) for our four treatments separately using multiple regression following Lande and Arnold (1983). These models control for correlations among traits and determine the targets of selection of the traits included. For the four treatments, we used relative fitness (seed number/treatment mean) as the dependent variable and variance standardized traits (mean zero and variance of one) as explanatory variables. Because some traits and fitness varied across cohorts, we included cohort as a block effect (mixed models were not used for the same reasoning as above but also produced very similar selection estimates). Our final models included traits representing plant size (height), phenology (days to flower), floral signals (display size, flower size), pollination efficiency (herkogamy), and reward (nectar volume). Petal length and flower size (diameter) were strongly correlated (see Results), so we only included flower size in the models. We used Pearson’s correlations to inform model inclusion of correlated traits and calculated variance inflation factors (VIFs) to ensure our final models were robust to issues of multicollinearity (all trait VIFs < 2). We tested for but found no evidence of non-linear selection. To assess whether models with fewer traits would provide more statistical power to detect patterns, we reduced models to include only traits with significant gradients (height, days to flower, and flower size). Similar patterns were observed but selection on days to flower in drought changed to *P < *.05. We present the full models because non-significant selection on traits is also informative, especially for rarely measured traits such as nectar.

To determine whether net (total) selection differed between water treatments, we analysed the open-pollinated plants with ANCOVA. We used the same base model with relative fitness (relativized within water treatment) as the response variable and the standardized (within water treatment) six phenotypic traits, water treatment, individual trait × water treatment interactions, and cohort as explanatory variables. A significant interaction between trait and water treatment was interpreted as selection differing with water availability. For this and other ANCOVA models, we only use the interaction terms because the main effects are uninformative.

We quantified pollinator-mediated selection as Δβ_poll_ = β_open_ – β_hand_ and the associated standard errors as $\sqrt{(SE_{\;\beta \;O}^{2}}+SE_{\;\beta \;H}^{2})$ separately for each watering treatment (Sletvold 2019). To test whether pollinator-mediated selection was significant within the water treatments, we first used separate ANCOVA models for each water treatment that included relative fitness (relativized within pollination treatment) as the response variable and the standardized six phenotypic traits, pollination, individual trait × pollination interactions, and cohort as explanatory variables. A significant trait × pollination treatment interaction indicated significant pollinator-mediated selection. To test whether pollinator-mediated selection differed with watering, we included all data and used ANCOVA. We used relative fitness (relativized within watering + pollination treatment) as the response variable and the standardized six phenotypic traits, pollination, water treatment, individual trait × water treatment × pollinator interactions, and cohort as explanatory variables. A significant trait × water treatment × pollinator treatment indicates pollinator-mediated selection differed with watering. We performed all analyses with R 4.3.0 (R Core Team 2021) in R studio (RStudio Team 2020), using the packages CAR v.3.0-10 (Fox *et al.* 2012), LME4 v.1.1-29 (Bates *et al.* 2015) and GGPLOT2 (Wickham 2016). For all models, we present the Type III *F*-tests or Type III Wald chisquare tests were appropriate.

## Results

### Trait expression and female fitness

We found an effect of both watering (Pillai’s Trace = 0.15, *F*_1,328_ = 6.26, *P* < .0001) and pollination treatment (Pillai’s Trace = 0. 088, *F*_1,328_ = 3.41, *P =* .0005) on our phenotypic traits and fitness in the MANOVA model. There was also a significant interaction (Pillai’s Trace = 0.055, *F*_1,328_ = 2.08, *P =* .031), suggesting that some aspects of the phenotypes were affected by the combination of treatments. Cohorts differed (Pillai’s Trace = 1.36, *F*_1,328_ = 17.28, *P* < .0001), so we retained the cohort within all subsequent analyses. Display size and both number of fruits and seeds were all reduced in drought compared to well-watered plants (Tables 1 and 2). For open-pollinated plants, herkogamy was increased in drought plants compared to well-watered, however, in hand-pollinated plants herkogamy was increased in well-watered plants. Interestingly, plants with supplemental pollination had more nectar and larger displays (Tables 1 and 2). Although many correlations were consistent across all our treatments, this was not always the case, especially when comparing between open pollination and supplemental hand pollination (Table 3). Flowering faster generally was associated with reduced peak flowering displays and, at least in well-watered conditions, smaller flowers. Petal length and overall flower size were strongly correlated but not perfectly so because diameter also depended on how petals were orientated and the width of the flower centre (Table 3).

**Table 1.** *Brassica rapa* phenotypic traits and female reproductive success (mean ± standard deviation) in well-watered and drought conditions combined with open pollination (OP) and supplemental hand pollination (HP) treatments.

Traits and fitness	Well-watered		Drought	
	OP	HP	OP	HP
Plant height (cm)	18.4 ± 4.1 (*N* = 95)	18.6 ± 4.8 (*N* = 93)	16.9 ± 4.3 (*N* = 90)	17.8 ± 4.0 (*N* = 85)
Days to flower	18.6 ± 2.2 (*N* = 96)	18.6 ± 1.9 (*N* = 94)	18.6 ± 2.1 (*N* = 91)	18.8 ± 2.4 (*N* = 85)
Display size	11.4 ± 5.0 (*N* = 95)	12.9 ± 6.8 (*N* = 93)	8.5 ± 4.4 (*N* = 90)	10.8 ± 5.1 (*N* = 84)
Petal length (mm)	3.52 ± 0.81 (*N* = 91)	3.63 ± 0.87 (*N* = 94)	3.42 ± 0.82 (*N* = 85)	3.45 ± 0.76 (*N* = 90)
Flower size (mm)	10.20 ± 1.63 (*N* = 91)	10.25 ± 1.60 (*N* = 94)	9.74 ± 1.56 (*N* = 85)	9.98 ± 1.62 (*N* = 90)
Herkogamy (mm)	1.35 ± 0.84 (*N* = 91)	1.60 ± 0.98 (*N* = 94)	1.47 ± 0.88 (*N* = 85)	1.24 ± 0.92 (*N* = 90)
Nectar volume (µl)	0.030 ± 0.022 (N = 100)	0.038 ± 0.031 (*N* = 99)	0.031 ± 0.026 (*N* = 96)	0.041 ± 0.029 (*N* = 98)
Fruits	10.8 ± 7.7 (*N* = 100)	8.2 ± 7.3 (*N* = 99)	5.5 ± 4.5 (*N* = 97)	5.8 ± 4.5 (*N* = 99)
Total seeds	88 ± 84 (*N* = 100)	66 ± 78 (*N* = 99)	40 ± 42 (*N* = 97)	40 ± 41 (*N* = 99)

**Table 2.** Statistics from generalized linear models testing the effects of watering, pollination, and their interaction on trait and fitness values, models are detailed in the text.

Traits and fitness	Water		Pollination		Water × Pollination	
	χ^2^	*P*	χ^2^	*P*	χ^2^	*P*
Plant height	2.73	.098	1.58	.21	0.45	.50
Days to flower	1.09	.29	0.65	.42	0.28	.60
Display size	**7.10**	**.0077**	**14.80**	**.00012**	3.40	.065
Petal length	2.28	.13	0.034	.85	0.50	.48
Flower size	1.19	.27	1.06	.30	0.39	.53
Herkogamy	**7.77**	**.0053**	3.27	.070	**8.10**	**.0044**
Nectar volume	0.41	.52	**6.95**	**.0084**	0.083	.77
Fruits	**10.33**	**.0013**	0.22	.64	**5.14**	**.023**
Total seeds	**12.30**	**.00045**	0.025	.87	2.80	.093

*P* < .05 are in bold.

**Table 3.** Pearson correlations among *Brassica rapa* traits in well-watered and drought conditions. Open-pollinated plants are above the diagonal and supplemented hand-pollinated plants are below for each water treatment.

Watered	Height	DTF	Display	Petal	Flw size	Herkogamy	Nectar
Height		0.011	0.210^*^	−0.152	−0.009	−0.126	−0.071
DTF	0.030		−0.231^*^	−0.080	−0.379^***^	0.366^***^	0.163
Display	−0.106	−0.389^***^		0.113	0.098	−0.007	−0.138
Petal	0.146	−0.033	−0.187		0.455^***^	0.178	0.007
Flw size	0.182	−0.137	−0.035	0.477^***^		−0.059	−0.190
Herkogamy	−0.117	0.062	−0.006	0.366^***^	0.072		−0.017
Nectar	−0.088	0.223^*^	−0.119	0.205*	−0.080	0.441^***^	

Drought	Height	DTF	Display	Petal	Flw size	Herk	Nectar
Height		−0.117	0.012	−0.109	0.118	0.011	0.021
DTF	−0.152		−0.301^**^	0.124	−0.071	0.251^*^	−0.002
Display	0.137	−0.371^***^		0.051	0.074	−0.107	0.094
Petal	0.225^*^	0.047	−0.077		0.432^***^	0.318^**^	−0.111
Flw size	0.247^*^	−0.105	0.126	0.457^***^		0.197	−0.078
Herkogamy	−0.255^*^	0.224^*^	−0.205	0.011	−0.253^*^		−0.165
Nectar	0.079	−0.223^*^	0.172	−0.214^*^	−0.023	−0.072	

DTF = days to flower, Petal = petal length, Flw size = flower size (diameter). Significant correlations highlighted in green for positive and red for negative.

^*^ *P* < .05,

^**^ *P* < .01,

^***^ *P* < .001.

### Pollen limitation and opportunity for selection

**Figure 2.**
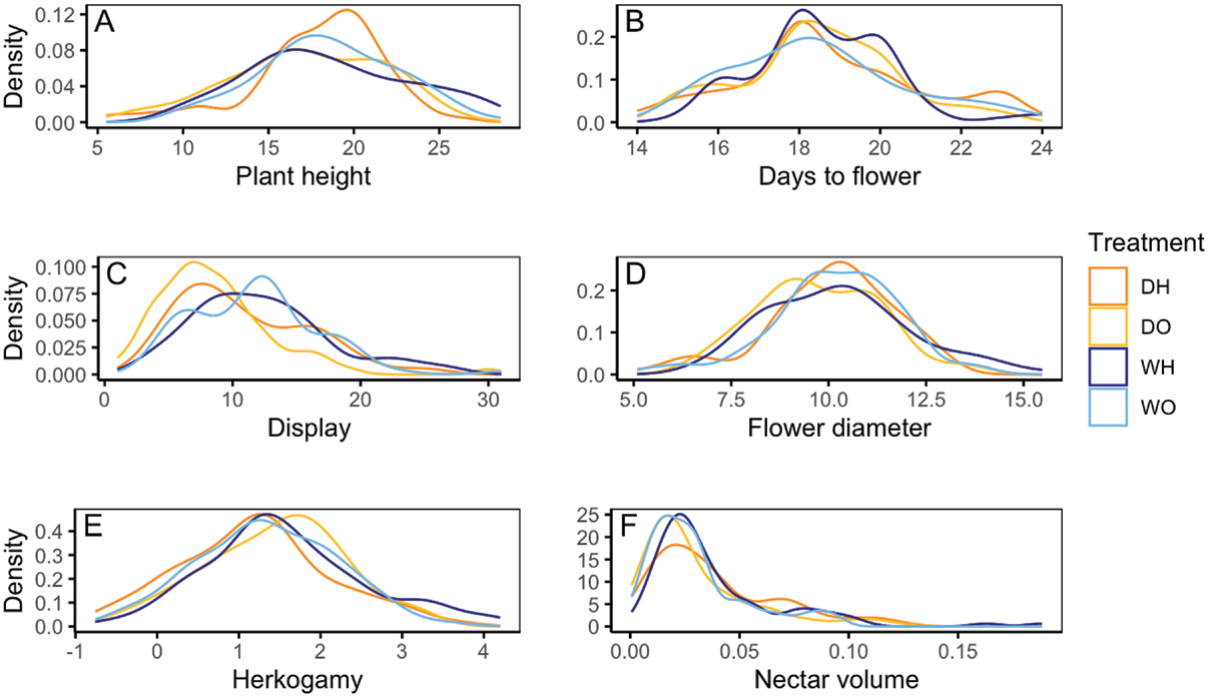
Density curves representing the distribution of *Brassica rapa* phenotypic trait values within four treatments. DH = drought and supplemental hand pollination, DO = drought and open pollination, WH = well-watered and supplemental hand pollination, WO = well-watered and open pollination.

Although we did not conduct systematic observations of visitation, we observed pollinators frequently visiting our plants (Fig. 1). Despite the restricted access to pollination, we found no evidence for pollen limitation in our experiment (Table 2). Under drought, plants had similar seed set and for well-watered plants supplemental hand-pollinated plants had fewer seeds than those with only pollinator visitation. We found the opportunity for selection was similar in well-watered plants (open-pollinated = 0.80, hand-pollinated = 1.27) as drought plants (open-pollinated = 0.90, hand-pollinated = 0.84), with supplemental pollination having little effect to reduce variance in relative fitness. The distributions of plant traits were also similar across the four treatments, suggesting trait distributions would not limit the ability to detect selection (Fig. 2).

### Phenotypic selection

**Figure 3.**
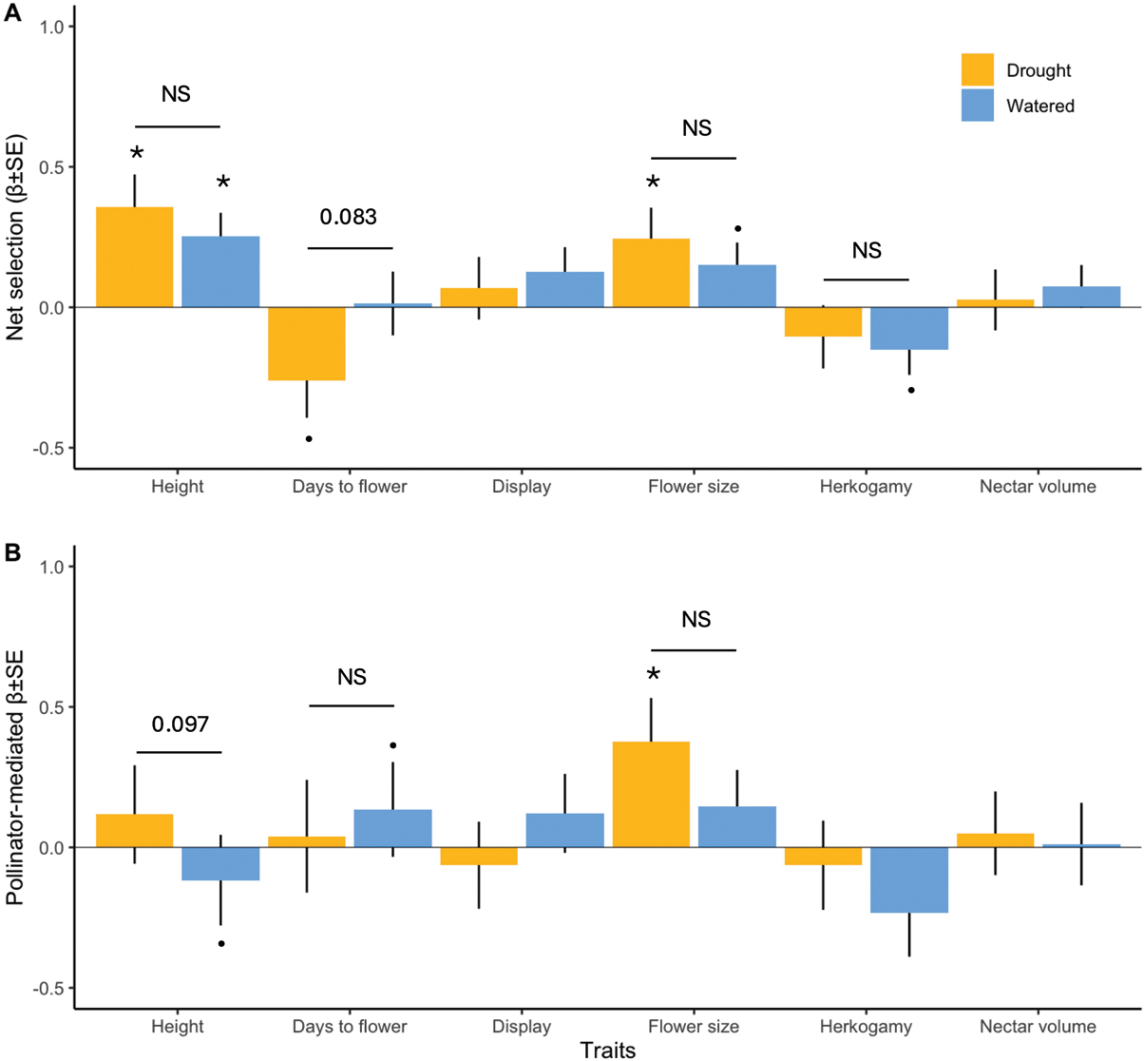
Phenotypic selection on plant height, days to flower, display size (number of flowers at peak flowering), flower size, herkogamy, and nectar volume in experimental populations of *Brassica rapa* grown in two soil water conditions (drought and well-watered). Net directional selection gradients were estimated from open-pollinated plants (a) and pollinator-mediated selection gradients were estimated as selection in open-pollinated plants—selection in hand-pollinated plants (b, Δβ_poll_ ± SE). Asterisks above bars indicate whether selection is significantly different from zero and the *P*-values above the lines indicate whether selection estimates differ between treatments from ANCOVA models. *P < .05,^.^*P* < .1, NS = non-significant, statistics in Table 4.

We found evidence for selection on plant height, days to flower, and flower size (Fig. 3), but in no context was there selection on display size, herkogamy, or nectar detected (Table 4). Selection for taller plants was found in both well-watered and drought conditions and was of similar strength. We found selection for fewer days to flower but only in drought (*P* = 0.051) and there was no strong evidence that selection on timing was pollinator-mediated. If anything, in well-watered plants there was marginally significant pollinator-mediated selection for flowering later rather than earlier (Fig. 3, Table 4). The interaction between days to flower and watering treatment was marginally significant (*P* = .083), suggesting the abiotic conditions were a driver of the net selection differences. We also found pollinator-mediated selection for bigger flowers in drought plants, but this did not differ significantly from well-watered plants which had a weaker non-significant selection gradient in the same direction (Fig. 3).

**Table 4.** Net selection statistics from linear models for open-pollinated plants run separately for well-watered and drought plants.

		Well-watered		Drought		Trait × Treatment	
	Traits	*F*_*1,78*_	*P*	*F*_*1,63*_	*P*	*F*_*1,145*_	*P*
Net selection	Plant height	**3.037**	**.003**	**3.044**	**.003**	0.29	.59
	DTF	0.12	.9	**−1.992**	**.051**	**3.06**	**.083**
	Display size	1.417	.16	0.61	.54	0.81	.37
	Flower size	**1.906**	**.06**	**2.209**	**.031**	0.33	.56
	Herkogamy	**−1.678**	**.097**	**−**0.933	.35	0.70	.40
	Nectar	0.983	.33	0.242	.81	0.52	.47
		*F*_*1,161*_	*P*	*F*_*1,137*_	*P*	*F*_*1,302*_	*P*
Pollinator-mediated selection	Plant height	**2.86**	**.093**	0.59	.44	**2.77**	**.097**
	DTF	**3.07**	**.082**	0.07	.79	1.53	.22
	Display size	0.67	.41	0.46	.50	1.12	.29
	Flower size	1.68	.20	**4.54**	**.035**	0.75	.39
	Herkogamy	1.05	.31	0.0003	.987	0.39	.53
	Nectar	0.10	.75	0.088	.77	0.46	.50

The traits × treatment tests whether selection differs from water treatment with an ANCOVA model. Pollinator-mediated selection statistics test whether selection differs between open and hand-pollinated plants and are from ANCOVAs with trait × pollination interactions done separately for each water treatment. The trait × treatment column represents the three-way interaction of trait × pollination × water from an ANCOVA of all data. See text for more details. *P*-values less than .10 are highlighted with bold.

## Discussion

As for many other species (Kuppler and Kotowska 2021), we found that water stress resulted in changes to the traits and fitness of *B. rapa*. Drought plants were generally smaller with smaller floral displays, that translated to reduced female fitness with fewer fruits and seeds then well-watered plants. However, despite these phenotypic changes, the drought did not have a large impact on the phenotypic selection of most *B. rapa* traits in our study. The most interesting result was finding a stronger selection for rapid flowering in drought conditions compared to well-watered. Our results mirror wild populations of *B. rapa* where natural drought has led to the evolution of earlier flowering (Franks *et al.* 2007) and changes to allele frequencies of genes related to flowering time (Franks *et al.* 2016). Artificial selection in drought conditions has also resulted in earlier flowering of *B. rapa* fast plants (Johnson *et al.* 2022). Early flowering may be an escape mechanism for *B. rapa*, allowing plants to complete their lifecycle quickly rather than evolving mechanisms to tolerate drought (Franks 2011). However, adaptation to drought maybe costly, for example, in terms of susceptibility to fungal infections in *B. rapa* (O’Hara *et al.* 2016). In our study, regardless of treatment, plants that flowered earlier also had fewer flowers at their peak flowering and in well-watered plants, faster flowering also was associated with smaller flowers suggesting trade-offs. Flowering time is an important life history trait, especially for annual plants which only make this transition to flowering once. Selection for early flowering is commonly found across many studies (Munguía-Rosas *et al.* 2011; Austen *et al.* 2017) and has been observed for other annual species in drought (Metz *et al.* 2020; Rauschkolb *et al.* 2023). We found no evidence that the selection for early flowering was pollinator-mediated and our results add to growing evidence that drought conditions can drive selection on flowering time independently of pollinator preferences.

We also found selection to increase flower size which was significant in drought but only marginally so in well-watered plants. While drought is often associated with reduced flower size (Carroll *et al.* 2001; Kuppler and Kotowska 2021; Kuppler *et al.* 2021), we did not find evidence of a response in our experimental conditions. However, a similar selection for bigger flowers in drought was seen in *Ipomea purpurea* (García *et al.* 2023). Larger flowers can be costly for plants under water stress (Galen 1999, 2000; Kuppler *et al.* 2021), which could suggest selection for smaller flowers in drought is more likely, however, thus far the only evidence for this pattern is from comparing two naturally different sites which could also vary in other ways (Wu *et al.* 2022). Selection to increase flower size is common (Harder and Johnson 2009) and often pollinator-mediated (Parachnowitsch and Kessler 2010; Trunschke *et al.* 2017). We also found that the magnitude of pollinator-mediated selection accounted for net selection on flower size, supporting the idea that pollinators often prefer and drive selection for larger flowers. Pollinator-meditated selection may be strong in stressful environments, such as drought, despite the costs of maintaining those flowers. However, our final sample size limited our ability to detect significant differences in the three-way interaction of comparing pollinator-mediated selection in well-watered and drought plants. Higher plant numbers and/or reducing pollinator visits to ensure pollen limitation may have led to more significant patterns.

We found the selection to increase height regardless of water availability, although more watering did lead to taller plants. Selection for taller plants can be common because it is often associated with plant vigour and/or plant apparency (Grindeland *et al.* 2005; Parachnowitsch and Kessler 2010; Zu and Schiestl 2017). For *B. rapa*, artificial selection on plant height has shown that it can evolve quickly and have correlated responses with other important traits for pollinator interactions such as floral volatiles, petal reflectance, and nectar sugars (Zu and Schiestl 2017). While plant height can be related to apparency, it is less likely in our potted experiment because there was no competing vegetation. For pollinators, display size can also act as an attractive trait by making the inflorescence more apparent but interestingly, unlike many other studies (Grindeland *et al.* 2005; Harder and Johnson 2009; Parachnowitsch and Kessler 2010; Sletvold and Ågren 2010), we did not find display size itself was a target of selection in our experiments. Neither height nor display had evidence for pollinator-mediated selection, suggesting that selection for increased height across our watering treatments was related to plant vigour.

Decreased nectar volumes are common with water stress (Carroll *et al.* 2001; Gallagher and Campbell 2017; Rering *et al.* 2020; García *et al.* 2023), however, we found no difference in nectar volumes with watering treatments in our *B. rapa* plants. We also did not find any evidence of net or pollinator-mediated selection on nectar. Few phenotypic selection studies have included nectar traits, making generalizations challenging (Parachnowitsch *et al.* 2019). However, one study with *B. rapa* found the selection to increase nectar volume, or at least on the principal component nectar loaded on, along with taller plants, more flowers, earlier flowering, and longer stamens (Dorey and Schiestl 2022). Another study comparing well-watered and drought conditions found selection to increase nectar in well-watered but decrease nectar in drought *Ipomea purpurea* (García *et al.* 2023), showing that water conditions can drive selection on nectar volumes. Our experiment highlights a challenge of drought treatments where it can be hard to determine a level of stress that causes phenotypic changes but does not stop flower production or lead to plant death.

Pollinators may detect nectar in *B. rapa* through attractive volatile compounds (Knauer and Schiestl 2015; Moreira *et al.* 2023) or other honest signals (Gervasi and Schiestl 2017). Unlike Gervasi and Schiestl (2017), we did not find plant height or flower size were correlated with nectar in open-pollinated plants and therefore were not honest signals. Although we detected selection on both traits, we cannot explain selection on either plant height or flower size because of their role as an honest signal of nectar rewards. Interestingly, we did find correlations between nectar and petal length in hand-pollinated plants but in the opposite direction depending on watering; well-watered plants had a positive relationship, while under drought it was negative. Supplemented pollination increased nectar production in our *B. rapa* suggesting a better understanding of dynamic nectar production in relation to pollination/flower handling could be important to understanding the full effects of pollination. Hand pollination did not affect nectar volume in the orchid *Platanthera chlorantha* (Atipiczynska 2003), but nectar removal can change replenishment (e.g. Castellanos *et al.* 2002) and other studies have shown nectar differences between unpollinated and pollinated flowers (Canto *et al.* 2011).

It is possible that our hand pollination was detrimental for the seed set, although this was only evident in well-watered plants that produced more seed overall. While we tried to provide a diversity of pollen to flowers and hand-pollinated plants also were exposed to natural pollination, flower handling may have caused reduced seed set or herbivore-like induced responses (Kessler and Chautá 2020). The lack of pollen limitation could have reduced our ability to detect pollinator-mediated selection (Benkman 2013), however, pollen limitation does not always predict selection (Sletvold and Ågren 2014). Pollinator-mediated selection can occur without pollen limitation if the relationship between trait values and fitness differs between pollination treatments (Galen 1996; Parachnowitsch and Kessler 2010). Our results of pollinator-mediated selection were consistent with other studies (e.g. on flower size [Parachnowitsch and Kessler 2010]), although it can be more common to detect stronger pollinator-mediated selection on efficiency traits such as herkogamy and plant-level attraction such as display size, rather than flower size as we saw (Caruso *et al.* 2019). Given our reduced seed set in hand-pollinated well-watered plants, the pollinator-mediated selection estimates for well-watered plants should be interpreted with caution.

Many floral traits likely represent adaptive compromises to selection by pollinator and non-pollinator agents of selection (Strauss and Whittall 2006). Selection mediated by abiotic factors such as soil water availability can be similar in strength to pollinator-mediated selection, although pollinator-mediated selection remains a focus of many studies and few manipulate more than one putative agent (Caruso *et al.* 2019; Sletvold 2019). Our factorial experiment allowed us to directly test the strength of selection imposed by biotic (pollinator) and abiotic (drought) agents of selection as well as how pollinator-mediated selection could depend on the abiotic context. Our study suggests that selection may be stronger in stressed conditions. However, it remains challenging to know the magnitude of each process that contributes to the selection observed in flowering plants (Strauss and Whittall 2006; MacColl 2011; Caruso *et al.* 2017). Conducting crossed-factor manipulative experiments such as ours to distinguish the effect of multiple agents of selection is critical to understand adaptive evolution in floral traits.

## Supplementary Material

plae070_suppl_Supplementary_Materials

## Data Availability

The data underlying this article are available in its online supplementary material.
